# Value of Follow-Up N-Terminal Probrain Natriuretic Peptide (NT-proBNP) after a Modified Fontan Procedure

**DOI:** 10.1155/2021/3300884

**Published:** 2021-10-15

**Authors:** Jianbin Li, Li Ma, Minghui Zou, Wenlei Li, Xinxin Chen, Yanqin Cui, Xiaoyan Hu

**Affiliations:** ^1^Heart Center, Guangdong Provincial Key Laboratory of Research in Structural Birth Defect Disease, Guangzhou Women and Children's Medical Center, Guangzhou Medical University, Guangzhou 510623, China; ^2^Department of Paediatrics, Guangdong Provincial Key Laboratory of Research in Structural Birth Defect Disease, Guangzhou Women and Children's Medical Center, Guangzhou Medical University, Guangzhou 510623, China; ^3^Department of Cardiac Surgery, Guangdong Provincial Key Laboratory of Research in Structural Birth Defect Disease, Guangzhou Women and Children's Medical Center, Guangzhou Medical University, Guangzhou 510623, China; ^4^Department of Nursing, Guangzhou Women and Children's Medical Center, Guangzhou Medical University, Guangzhou 510623, China

## Abstract

**Objective:**

To assess the value of N-terminal probrain natriuretic peptide (NT-proBNP) in short-term and long-term follow-up after a modified Fontan procedure.

**Methods:**

We retrospectively enrolled children who had undergone a modified Fontan procedure in the Heart Center of Guangzhou Women and Children's Medical Center from January 2014 to September 2020 and collected data on NT-proBNP values before bidirectional Glenn procedure, before Fontan procedure, and on 1, 3, 7, 30, 90, and 180 days and 1, 2, 3, 4, 5, and 6 year after Fontan procedure. The relationship between changes in NT-proBNP levels and the outcomes in children was analyzed.

**Results:**

A total of 108 children (78 boys and 30 girls, mean age: 54.62 ± 29.38 weeks) were included in the analysis. According to one-way analysis of variance, the left ventricular type and biventricular type of single ventricle physiology showed shorter duration on cardiopulmonary bypass during the operation and lower levels of NT-proBNP after the operation than the right ventricular type and univentricular type physiology.

**Conclusion:**

NT-proBNP is a good indicator for mid and long-term follow-up after a modified Fontan procedure. The left ventricular type and biventricular type of single ventricle physiology show better mid and long-term benefits from the modified Fontan procedure than the right ventricular type and univentricular type physiology.

## 1. Introduction

The modified Fontan procedure is a final palliative procedure for treating complex congenital heart diseases such as anatomical or functional single ventricle (e.g., tricuspid atresia, mitral atresia, and hypoplastic left heart syndrome) that are not suitable for biventricular correction [1]. The Fontan operation aims to eliminate the symptoms of hypoxia and reduce the volume load of the single ventricle. It has evolved from atriopulmonary connection to total cavopulmonary connection. Moderately elevated venous pressure and sucking power of the functional ventricle are critical for patients palliated using a Fontan procedure. Abnormally increased pressure in the vena cava or decreased cardiac output can lead to chronic heart failure in patients after Fontan. Therefore, monitoring cardiac function after Fontan is of great significance to ensure good quality of life and facilitate timely intervention.

N-terminal probrain natriuretic peptide (NT-proBNP) is a marker for cardiac volume load. It is synthesized and secreted in the ventricle and released in response to the volume and pressure overload of the heart. NT-proBNP has been demonstrated to be useful in the diagnosis and treatment of heart failure [2, 3]. However, to our knowledge, only limited data are available regarding the perioperative and postoperative values of NT-proBNP in the diagnosis of heart failure and the follow-up of outcomes. In this retrospective study of patients undergoing Fontan procedures, we investigated the changes in NT-proBNP over the perioperative period and long-term postoperative follow-up period, analyzed the relationship between NT-proBNP and echocardiographic parameters during follow-up, and discussed the value of NT-proBNP in the postoperative follow-up of cardiac function in children with single ventricle physiology.

## 2. Materials and Methods

This retrospective study was approved by the Ethics Committee of Guangzhou Women and Children's Medical Center. All hospitalized patients who had undergone a modified Fontan procedure (intracardiac or extracardiac conduit) with cardiopulmonary bypass in our hospital from January 2014 to September 2020 were eligible. Patients lost to follow-up were excluded. Patients were included when they had regular clinic and telephone follow-ups and had regular NT-proBNP tests and echocardiographic examinations. NT-proBNP detection principle is the electrochemiluminescence method, and we used the Roche E 601 analyzer to complete the detection. A total of 108 cases were included in the analysis. The collected clinical data included demographic features, staged anatomical features of the major blood vessels of the heart, surgical and cardiopulmonary bypass records, and follow-up data. The detailed patient data are given in Table 1.

All cases were classified into left ventricular type, right ventricular type, biventricular type, and univentricular type according to the functional ventricular morphology of congenital heart anomalies (Table 2). The classification of single ventricle was based on the findings of cardiac echo. If there were clear left and right ventricular structures and corresponding valve structures, the children were classified as biventricular type, for example, DORV and TGA/VSD/PS; if there were only fused single atrioventricular valves and the ventricles could not be distinguished, the children were classified as univentricular type. If there were mitral valve or tricuspid valve atresia or one ventricle obviously dysplasia, the patients were classified as left or right ventricular type, and the relevant statistical analysis results are given in Table 3.

During our follow-up, 1 case with right ventricular type developed progressive cardiac failure, 7 cases developed cardiac arrhythmias after surgery, including 4 patients with paroxysmal upper cardiac tachycardia, 1 patient with atrioventricular block requiring pacemaker placement, and 2 patients with ventricular arrhythmias. Significant proteinolytic enteropathy was found in 3 patients, and superior vena cava thrombosis was found in 1 patient. No pulmonary arteriovenous fistula, renal failure, and Fontan postoperative-associated liver disease were found; one patient died shortly after Fontan's operation, and no death or reoperation was found during the follow-up.

## 3. Discussion

The hemodynamics after a modified Fontan procedure is considerably different from that of normal biventricular physiology, leading to markedly increased long-term mortality and dysfunction in patients who have undergone Fontan operation. It has been demonstrated that the increased mortality rate after operation is mainly caused by heart failure, arrhythmia, protein-losing enteropathy, pulmonary arteriovenous fistula, thrombosis, renal failure, and Fontan-related nephropathy; thus, many indicators have been suggested to monitor Fontan circulation during follow-up [4].

NT-proBNP, an inactive prohormone of brain natriuretic peptide, is secreted by cardiac muscle tissue in response to abnormal volume load or pressure load of the ventricle wall [5, 6]. BNP has important pathophysiological significance. It can promote the excretion of sodium and urine, has a strong vasodilator effect, and can resist the vasoconstriction of the renin angiotensin aldosterone system (RAAS) [7]. Moreover, BNP is further affected by oxidative stress and mitochondrial homeostasis/calcium homeostasis [8, 9]. It is a recognized good indicator for assessing ventricular dysfunction or heart failure. However, there are few studies on the relationship between NT-proBNP and cardiac function, outcomes, and relevant influencing factors in congenital heart disease, especially in single ventricle physiology.

A study of 77 cases by Lowenthal et al. reported the normal upper limit values of NT-proBNP in different palliation stages of single ventricle physiology: 300 pg/mL after a Blalock–Taussig shunt or pulmonary banding, 1100 pg/mL after bidirectional Glenn operation, and 1900 pg/mL after Fontan operation. The researchers noted that NT-proBNP is a sensitive and effective tool to evaluate cardiac function changes after Fontan operation.

With a larger number of cases, our study showed a good correlation between NT-proBNP and mid and long-term cardiac functions after a modified Fontan procedure. The changes in NT-proBNP levels showed high sensitivity in reflecting the changes in cardiac function of children, especially in those with postoperative valve reflux, arrhythmia, and residual pulmonary venous obstruction, whose NT-proBNP levels were significantly increased. Our results were consistent with Chen's findings that severe valve reflux and pulmonary arterial pressure of >15 mmHg are risk factors for heart failure and show poor outcome after a modified Fontan procedure [10, 11].

Comparison of the mid and long-term post-Fontan outcomes between different types of single ventricle physiology in our study showed that cases of left ventricular type had a relatively good long-term prognosis, and surprisingly, cases of biventricular type showed relatively good mid and long-term outcomes after Fontan operation, who would, in theory, be expected to benefit more from biventricular correction. However, prior to our study, there has been little research about the mid and long-term outcomes after modified Fontan operation in complex congenital heart disease such as double outlet right ventricle with noncommitted ventricular septal defect. The significant differences in cardiopulmonary bypass time during operation, blood oxygen saturation after operation, and NT-proBNP levels at 7 days, 30 days, and 180 days after operation indicated that left ventricular type and biventricular type physiology were associated with shorter operation time, more rapid recovery of cardiac function, and more normal blood oxygen saturation after a modified Fontan procedure than univentricular type and right ventricular type physiology. The possible reason for the poor prognosis in the right ventricular group was that the right ventricular structure and tricuspid valve could not tolerate the high afterload of systemic circulation for a long time, leading to the symptoms of gradually worsening heart failure.

There were no significant differences between left ventricular type and biventricular type with respect to echocardiographic results and NT-proBNP values after Fontan operation, suggesting no significant difference in mid and long-term outcomes between the two types of single ventricle physiology. The possible reason for the difference of blood flow velocity in upper and lower lumens after modified Fontan is that the abnormal increase of pulmonary circulation resistance causes the obstruction of blood flow in upper and lower lumens, and the decline of ventricular diastolic function and valve regurgitation are the main reasons for the increase of pulmonary circulation resistance. However, this finding is limited by the small number of patients in this study, and hence, more cases should be enrolled and analyzed in future studies to validate and improve this research.

## 4. Conclusion

This mid and long-term follow-up study found that NT-proBNP is a sensitive and reliable indicator to evaluate cardiac function after a modified Fontan procedure. The left ventricular type and biventricular type of single ventricle physiology show better outcomes after operation in terms of valve reflux, blood oxygen saturation, and blood flow velocity of the superior and inferior vena cava than the right ventricular type and univentricular type.

## Figures and Tables

**Table 1:** General clinical information.

Terms	Cases
Sex (male/female)	78/30
Age at operation (weeks)	54.62 ± 29.38
Body height (cm)	97.46 ± 16.74
Body surface area (m^2^)	0.67 ± 0.52
Saturation of peripheral oxygen (%)	76.33 ± 13.65
Preoperative hematocrit	47.48 ± 6.79
Proximal *M* value	2.42 ± 0.71
Distal *M* value	2.13 ± 0.65
Nakata index (mm^2^/m^2^)	335.9 ± 201.1
Pulmonary vascular resistance after correction (wood)	2.65 ± 1.0
18# extracardiac conduit (number of cases)	23
20# extracardiac conduit (number of cases)	59
22# extracardiac conduit (number of cases)	9
30# extracardiac conduit (number of cases)	1
Fenestration (number of cases)	101
Duration of cardiopulmonary bypass (minute)	112.77 ± 49.15
Aortic clamping (number of cases)	9
Median duration of aortic clamping (minute)	79
Duration of postoperative assisted ventilation (hour)	6
Stay in cardiac intensive care unit (day)	3
Postoperative blood oxygen saturation	90

Proximal *M* value: (proximal left pulmonary artery diameter plus proximal right pulmonary artery)/descending aorta diameter at diaphragm level. Distal *M* value: (distal left pulmonary artery diameter plus distal right pulmonary artery)/descending aorta diameter at diaphragm level.

**Table 2:** The classification characteristics of patients admitted for modified Fontan procedure.

Type of single ventricle		Number of cases
Left ventricular type	Tricuspid atresia (TA)	21
	Pulmonary atresia with intact ventricular septum (PA/IVS)	2
	Pulmonary atresia with ventricular septal defect (PA/VSD)	1
	Double inlet left ventricle (DILV)	3
	Severe pulmonary stenosis (PS)	4
	Heterotaxy syndrome	1

Right ventricular type	Double inlet left ventricle (DILV)	1
	Double inlet right ventricle (DIRV)	3
	Heterotaxy syndrome	10
	Unbalanced complete atrioventricular septal defect (UBCAVC)	3
	Mitral atresia (MA)	2

Biventricular type	Transposition of the great arteries with ventricular septal defect and pulmonary stenosis (TGA/VSD/PS)	6
	Double outlet right ventricle with ventricular septal defect and pulmonary stenosis (DORV/VSD/PS)	8
	Congenitally corrected transposition of the great arteries with ventricular septal defect and pulmonary stenosis (ccTGA/VSD/PS)	10
	Congenitally corrected transposition of the great arteries with ventricular septal defect and pulmonary atresia (ccTGA/VSD/PA)	5
	Transposition of the great arteries with ventricular septal defect and pulmonary atresia (TGA/VSD/PA)	5
	Transposition of the great arteries with ventricular septal defect and pulmonary hypertension (TGA/VSD/PH)	1
	Congenitally corrected transposition of the great arteries with ventricular septal defect and pulmonary hypertension (ccTGA/VSD/PH)	1
	Double outlet right ventricle with ventricular septal defect and pulmonary hypertension (DORV/VSD/PH)	2
	Double outlet right ventricle with left ventricular outflow tract obstruction (DORV/LVOTO)	1

Univentricular type	Double inlet left ventricle (DILV)	2
	Heterotaxy syndrome	7
	Unbalanced complete atrioventricular septal defect (UBCAVC)	5
	Double inlet right ventricle (DIRV)	1
	Transposition of the great arteries with ventricular septal defect and pulmonary atresia (TGA/VSD/PA)	1

**Table 3:** Analysis of the difference among the patients admitted for modified Fontan procedure.

Group	Significantly different variables	Mean difference	*P* value
Left ventricular type vs. univentricular type	NT-proBNP level before Fontan operation	−26.41	0.015
Right ventricular type vs. biventricular type	Blood flow velocity of the left superior vena cava 1 day after operation	−0.2912	0.01
Univentricular type vs. biventricular type	Blood flow velocity of the left superior vena cava on postoperative day 1	−0.2428	0.049
Left ventricular type vs. univentricular type	Blood flow velocity at the anastomosis of the pulmonary artery and conduit on postoperative day 1	−0.104	0.002
Right ventricular type vs. biventricular type	Blood flow velocity at the anastomosis of the pulmonary artery and conduit on postoperative day 1	−0.151	0.00
Right ventricular type vs. biventricular type	Blood flow velocity of the left superior vena cava 6 months after the operation	−0.16	0.017
Right ventricular type vs. left ventricular type	Blood flow velocity of the inferior vena cava 1 year after the operation	−0.075	0.019
Left ventricular type vs. right ventricular type	Valve reflux 1 year after the operation	−0.845	0.02
Left ventricular type vs. right ventricular type	Blood flow velocity of the right superior vena cava 2 years after the operation	−0.116	0.022
Right ventricular type vs. biventricular type	Blood flow velocity of the inferior vena cava 2 years after the operation	−0.082	0.03
Right ventricular type vs. biventricular type	Blood flow velocity of the right superior vena cava 3 years after the operation	−0.089	0.034
Left ventricular type vs. biventricular type	Blood flow velocity of the inferior vena cava 3 years after the operation	−0.078	0.016
Left ventricular type vs. right ventricular type	Blood flow velocity of the right superior vena cava 4 years after the operation	0.17	0.014
Left ventricular type vs. right ventricular type	Blood flow velocity at the anastomosis of the pulmonary artery and conduit 4 years after the operation	0.26	0.00
Left ventricular type vs. univentricular type	Blood flow velocity at the anastomosis of the pulmonary artery and conduit 4 years after the operation	0.23	0.00
Left ventricular type vs. biventricular type	Blood flow velocity at the anastomosis of the pulmonary artery and conduit 4 years after the operation	0.11	0.001
Univentricular type vs. biventricular type	Blood flow velocity at the anastomosis of the pulmonary artery and conduit 4 years after the operation	−0.11	0.002
Right ventricular type vs. left ventricular type	NT-proBNP level 7 days after the operation	1143.95	0.003
Right ventricular type vs. biventricular type	NT-proBNP level 7 days after the operation	1007.51	0.007
Right ventricular type vs. univentricular type	NT-proBNP level 7 days after the operation	942.29	0.038
Right ventricular type vs. biventricular type	NT-proBNP level 30 days after the operation	42.96	0.023
Left ventricular type vs. right ventricular type	NT-proBNP level 180 days after the operation	−872.59	0.001
Right ventricular type vs. univentricular type	NT-proBNP level 180 days after the operation	857.032	0.014
Right ventricular type vs. univentricular type	NT-proBNP level 180 days after the operation	1008.84	0.000
Left ventricular type vs. right ventricular type	Duration of cardiopulmonary bypass	−27.01	0.049
Left ventricular type vs. univentricular type	Duration of cardiopulmonary bypass	−47.34	0.001
Univentricular type vs. biventricular type	Duration of cardiopulmonary bypass	49.08	0.001
Right ventricular type vs. biventricular type	Duration of cardiopulmonary bypass	28.74	0.032
Left ventricular type vs. univentricular type	Duration of aortic clamping	−29.19	0.002
Right ventricular type vs. univentricular type	Duration of aortic clamping	−20.18	0.043
Univentricular type vs. biventricular type	Duration of aortic clamping	27.26	0.002
Left ventricular type vs. univentricular type	Blood oxygen saturation at discharge	15.17	0.019
Univentricular type vs. biventricular type	Blood oxygen saturation at discharge	−13.99	0.033

## Data Availability

The raw clinical data cannot be provided due to privacy concerns, but will have the data available once permission is obtained from the corresponding author or institution.
